# Predicting influenza in the post-COVID era: assessing LSTM, GRU, and transformer robustness to covariate shift

**DOI:** 10.3389/frai.2026.1886896

**Published:** 2026-08-11

**Authors:** Atiqa Naeem Alam Din, Woldegebriel Assefa Woldegerima, Jianhong Wu

**Affiliations:** 1 Disease-Informed Modeling, Methods & Systems (DIMMS) Lab, Department of Mathematics and Statistics, York University, Toronto, ON, Canada; 2 Laboratory for Industrial and Applied Mathematics (LIAM), Department of Mathematics and Statistics, York University, Toronto, ON, Canada; 3 Centre of Excellence in Artificial Intelligence for Public Health, York University, Toronto, ON, Canada

**Keywords:** attention mechanism, back propagation, GRU (Gated Recurrent Unit), influenza, LSTM (Long Short Term Memory Networks), regime shift, transformer

## Abstract

Forecasting influenza has become increasingly challenging due to post-COVID disruptions in seasonality and strain circulation. This work compares the performance of Long Short Term Memory Networks (LSTM), Gated Recurrent Unit (GRU), and transformer models in forecasting influenza spread using multivariate epidemiological and environmental data, with a focus on robustness under post-COVID non-stationarity. We compare LSTM, GRU, and transformer architectures within a multivariate deep learning framework using influenza and temperature data from Ontario (2014–2025), with data split into training, validation, and testing periods. Although recurrent models outperform transformers on limited, noisy data, all architectures exhibit marked performance collapse under post-COVID non-stationarity. The GRU and LSTM track pre-COVID seasonal peaks more closely, yet both substantially under-estimate the post-COVID resurgence, indicating that none of the models generalize across the regime shift. These findings position our study as a diagnostic of how architectural inductive biases break down under covariate shift. Furthermore, this manuscript assesses how the COVID-19 pandemic affected the accuracy and performance of machine learning algorithms and notes the integration of transfer learning and attention mechanisms to improve model performance.

## 1 Introduction

Influenza, commonly known as flu, is a respiratory infectious disease that is highly contagious. It is caused by Influenza viruses that belong to the Orthomyxoviridae family. Influenza viruses are classified into four types: Influenza A, Influenza B, Influenza C and Influenza D (Lagace-Wiens et al., 2010). Influenza A and B are the cause of seasonal epidemics in humans. Symptoms of influenza include runny nose, fever, cough, sore throat, headache, muscle aches, and, in severe cases, breathing problems and pneumonia that may be fatal (World Health Organization, 2025). Transmission occurs mainly through droplets from coughing or sneezing or by coming in contact with infected surfaces. Symptoms develop 1–4 days after infection and usually last around a week (World Health Organization, 2025; Government of Canada, Public Health Agency of Canada, 2026). Some people are at a higher risk of infection, including children under 5 years of age, pregnant women, adults above 65 years of age, and people with chronic illnesses (World Health Organization, 2025; Government of Canada, Public Health Agency of Canada, 2026). Influenza virus has a single-stranded negative-sense RNA genome (World Health Organization, 2010). The hemagglutinin (HA) and neuraminidase (NA) glycoproteins are spread over the surface of the virus (World Health Organization, 2010). The HA protein binds with sialic acid in the human body to fuse inside the cell membrane, while the NA protein cleaves sialic acid to facilitate the release of the virus from the cell (World Health Organization, 2010). The replication cycle is rapid, with newly replicated viruses exiting infected cells within approximately 6 h. In influenza A and B viruses, the RNA genome is divided into 8 segments (Lagace-Wiens et al., 2010). The divided segments are involved in the genetic reassortment of the virus. The antigenic shift in the virus brings new strains that can cause pandemic threat if the new strain is more virulent or more resistant (Lagace-Wiens et al., 2010).

According to the World Health Organization (2025), influenza causes one billion infections and 290,000 to 650,000 deaths worldwide annually. In Canada, an average of 12,200 hospitalizations and 3,500 influenza-related deaths occur each year (Public Health Agency of Canada, 2016). Vaccination remains the most effective preventive measure, particularly for high-risk groups (World Health Organization, 2025; Government of Canada, Public Health Agency of Canada, 2026). While most individuals recover without treatment, severe cases may require antiviral medications (Government of Canada, Public Health Agency of Canada, 2026). Historically, influenza has caused several major pandemics, including the 1918 H1N1, 1957 H2N2, and 1968 H3N2 pandemics, as well as the 2009 H1N1 pandemic (Lagace-Wiens et al., 2010).

Accurate influenza forecasting is essential for public health planning, resource allocation, and early intervention. However, influenza dynamics are shaped by complex interactions among viral evolution, population immunity, environmental factors, and behavioral patterns. These factors introduce strong seasonality, non-stationarity, and abrupt regime shifts, particularly evident in the post-COVID-19 period, in making predictions.

Recent advances in deep learning have shown promise for epidemic forecasting. Recurrent neural networks (RNNs), including Long Short-Term Memory (LSTM) and Gated Recurrent Unit (GRU) architectures, have demonstrated strong performance in modeling temporal dependencies in infectious disease data (Venna et al., 2018; Alzamel, 2025). More recently, transformer models, which work on self-attention mechanism, have achieved state-of-the-art results in many sequence-modeling tasks (Prakash et al., 2025; Li et al., 2021). Despite this progress, few studies have systematically compared RNN-based and transformer-based architectures for real-world influenza forecasting, particularly under conditions of limited data and non-stationary epidemic behavior.

In this study, we develop a multivariate deep learning framework for influenza forecasting that integrates epidemiological data with environmental variable, i.e., temperature, to better capture seasonal and climatic patterns. We perform a systematic comparison of the LSTM, GRU and transformer architectures using real surveillance data from Ontario, Canada, evaluating how architectural differences influence the accuracy, stability, and sensitivity of the forecast to non-stationary epidemic behavior. By examining model performance across pre- and post-COVID periods, we analyze the robustness of recurrent and attention-based models under regime shifts.

This study contributes a baseline-anchored robustness diagnosis of modern sequence models under the profound regime shifts introduced by COVID-19. Unlike prior comparative influenza forecasting studies (Agyemang, 2025), which evaluate models exclusively in pre-COVID settings, we analyze both pre- and post-COVID periods to quantify how LSTM, GRU, and transformer architectures break down when trained on stable historical patterns and tested on structurally altered dynamics. Using Ontario's influenza data, which uniquely captures the multi-year collapse and re-emergence of seasonal circulation, we conduct a controlled architecture-level comparison with unified preprocessing, identical input windows, consistent data splits, and training conditions. We further integrate temperature as an exogenous driver and assess its contribution through ablation experiments, and we benchmark deep learning models against classical baselines to contextualize their failure modes. Together, these elements provide a systematic evaluation of model fragility under post-COVID non-stationarity in a small-sample epidemiological setting.

The rest of this paper is structured as follows. Section 2 reviews related work on existing influenza prediction approaches. Section 3 details the methodology and model architectures. Section 4 analyzes and compares the results of the different models. Finally, Section 5 concludes the paper and outlines future work.

## 2 Related works

Deep learning has become an increasingly prominent tool in infectious disease forecasting, driven by the availability of large-scale surveillance data and advances in sequence modeling. Early work demonstrated that recurrent neural networks (RNNs) and their gated variants outperform traditional statistical approaches such as ARIMA and exponential smoothing for influenza prediction (Venna et al., 2018; Zhang and Nawata, 2017). Comprehensive reviews highlight the growing role of deep learning and big data in epidemic forecasting, emphasizing the ability of neural architectures to capture nonlinear temporal dependencies and complex seasonal patterns (Chae et al., 2018).

Beyond recurrent models, convolutional neural networks (CNNs) and temporal convolutional architectures have also been applied to epidemic prediction, showing competitive performance in capturing local temporal structure and short-term dynamics (Lee et al., 2021). More recent studies have explored hybrid approaches that incorporate web-search trends, mobility data, or environmental variables to improve predictive accuracy and uncertainty estimation (Morris et al., 2023). These works highlight the importance of multivariate inputs in modeling influenza transmission.

Transformer-based architectures have gained significant attention due to their ability to model long-range dependencies through self-attention. In the context of epidemiology, transformers have been applied to influenza forecasting and COVID-19 prediction, demonstrating strong multi-step forecasting performance when sufficient training data are available (Li et al., 2021). Attention mechanisms have also been integrated into LSTM models to enhance interpretability and improve performance in small region COVID-19 forecasting (Hu et al., 2024). However, despite their success in other domains, transformers remain underexplored in real-world influenza forecasting, particularly in settings characterized by limited data, strong seasonality, and non-stationary epidemic behavior.

While prior studies such as Amendolara et al. (2023) have demonstrated the effectiveness of LSTM-based models for influenza forecasting, and Li et al. (2021) have explored attention mechanisms for epidemic prediction, these works typically evaluate a single architecture or focus on short-term, pre-pandemic regimes. The work of Agyemang (2025) presents a comprehensive comparative study of influenza forecasting, evaluating classical statistical models alongside deep learning architectures such as LSTM, GRU, and transformers. Their analysis focused on identifying the best-performing model under stable seasonal dynamics, using influenza data from the Centers for Disease Control and Prevention (CDC), USA, and optimizing hyperparameters to improve short-term prediction accuracy. While their results highlight the advantages of recurrent neural networks over traditional baselines, the study is limited to pre-COVID influenza patterns and does not examine how model performance changes under structural regime shifts. Moreover, their models were tuned independently, making it difficult to isolate architectural effects from differences in hyperparameter choices. In contrast, our work extends this line of research by evaluating LSTM, GRU, and Transformer models under a unified experimental setting and by explicitly assessing model behavior across the post-COVID regime shift, where influenza dynamics deviate substantially from historical norms. This distinction allows our study to diagnose robustness and failure modes that are not addressed in the previous studies. We use influenza cases in Ontario from January 2014 to November 2025 spanning both pre- and post-COVID periods, capturing the collapse and re-emergence of seasonal circulation. We provide an explicit evaluation of model generalization under post-COVID regime shifts, quantifying how architectures trained on stable historical patterns deteriorate when exposed to structurally altered dynamics. We also integrate temperature as an exogenous variable and assess its contribution through controlled ablation experiments, extending beyond the univariate influenza-only frameworks common in earlier work. Finally, we conduct a controlled architecture-level comparison, training all deep learning models with identical batch sizes, number of epochs, training windows, optimizers, loss functions, layer depths, and dropout rates to isolate architectural inductive biases. Hence, our work addresses a critical gap in understanding how modern sequence models generalize under non-stationary epidemic conditions.

## 3 Preliminary mathematical theory

Artificial Neural Networks (ANNs) are computational systems inspired by biological neural processing, consisting of interconnected artificial neurons arranged in layers (Yadav, Yadav; Chakraverty and Mall, 2017). While feed-forward architectures process information in a single direction, sequential data require mechanisms to retain information over time, motivating recurrent and attention-based architectures.

Epidemic time-series forecasting requires models capable of learning temporal dependencies from historical observations to predict future disease incidence. Neural sequence models such as LSTM, GRU and transformers provide flexible function approximators that capture nonlinear, seasonal, and long-range temporal structure in epidemiological data.

We select the LSTM, GRU, and transformer architectures because they represent three widely used modeling frameworks for time-series prediction. LSTMs remain the standard for epidemiological and clinical time-series forecasting due to their ability to capture long-range temporal dependencies (Hochreiter and Schmidhuber, 1997; Venna et al., 2018). GRUs offer a simplified recurrent structure with fewer parameters and often outperform LSTMs on short or noisy datasets (Cho et al., 2014). Transformers replace recurrence with self-attention, enabling flexible non-local dependency modeling and achieving state-of-the-art performance in many sequence domains (Vaswani et al., 2017; Lim and Zohren, 2021).

### 3.1 Recurrent neural networks

Given an input sequence (*x*_1_, *x*_2_, …, *x*_*T*_), a recurrent neural network maintains a hidden state *s*_*t*_ that summarizes past information. At each time step,


$$\begin{array}{l} s_{t}=f\left(W_{ss}s_{t-1}+W_{sx}x_{t}+b_{s}\right),\\ y_{t}=g\left(W_{ys}s_{t}+b_{y}\right), \end{array}$$

where *W*_*ss*_, *W*_*sx*_, *W*_*ys*_ are weight matrices, *b*_*s*_, *b*_*y*_ are biases, and *f* is typically tanh or ReLU. The hidden state acts as a memory of all previous inputs.

#### 3.1.1 Training via backpropagation through time and loss functions

Training a recurrent or sequence model minimizes a sequence loss *L*(*y*_1_, …, *y*_*T*_) by propagating gradients backward through the unrolled network across time steps (backpropagation through time, BPTT). For a decomposable loss $L=\overset{T}{\underset{t=1}{\sum }}\ell_{t}$, the gradient with respect to the recurrent weight matrix *W*_*ss*_ is


$$\begin{array}{l} \frac{\partial L}{\partial W_{ss}}=\overset{T}{\underset{t=1}{\sum }}\frac{\partial \ell_{t}}{\partial s_{t}}\frac{\partial s_{t}}{\partial W_{ss}}. \end{array}$$

Because the hidden state *s*_*t*_ depends recursively on previous states, we have


$$\begin{array}{l} \frac{\partial s_{t}}{\partial W_{ss}}=\frac{\partial s_{t}}{\partial s_{t-1}}\frac{\partial s_{t-1}}{\partial W_{ss}}=\left(\overset{t}{\underset{i=t-k+1}{\prod }}\frac{\partial s_{i}}{\partial s_{i-1}}\right)\frac{\partial s_{t-k}}{\partial W_{ss}}. \end{array}$$

The repeated product of jacobians


$$\begin{array}{l} \overset{t}{\underset{i=t-k+1}{\prod }}\frac{\partial s_{i}}{\partial s_{i-1}} \end{array}$$

is the mechanism by which error signals propagate through time. When the activation function derivatives are small (as with tanh or sigmoid), these products shrink exponentially, causing the *vanishing gradient problem*. Conversely, if the derivatives are large, gradients may explode.

The choice of the loss function directly shapes the error signals that BPTT distributes across time. Depending on the prediction task and the scale of the target variable, different loss functions are used. In our work, we use mean squared error (MSE) loss, with weighting applied. We now specify the loss function we used to train all the three deep learning models (LSTM, GRU and transformer) in our work.

Mean squared error (MSE) For a sequence of length *T*, with true values *y*_*t*_ and predictions ŷ_*t*_, the mean squared error is


$$\begin{array}{l} L_{\text{MSE}}=\frac{1}{T}\overset{T}{\underset{t=1}{\sum }}{\left(y_{t}-{ŷ}_{t}\right)}^{2}. \end{array}$$

Standard scaler. In this work, we use the standard scaler, which transforms each value *y*_*t*_ as


$$\begin{array}{l} y_{t}^{\text{scaled}}=\frac{y_{t}-\mu }{\sigma }, \end{array}$$ (1)

where μ and σ are the mean and standard deviation computed on the training set. The inverse transform is


$$\begin{array}{l} S^{-1}\left(y_{t}^{\text{scaled}}\right)=\sigma y_{t}^{\text{scaled}}+\mu . \end{array}$$

Weighted MSE To allow different time points to contribute unequally to the training objective, we use a weighted mean squared error of the form


$$L=\frac{1}{T}\sum_{t=1}^{T}w_{t}{\left(S^{-1}(y_{t})-S^{-1}({\hat{y}}_{t})\right)}^{2},$$

where *w*_*t*_ ≥ 0 is a per-time weight and $S^{-1}$ denotes the inverse of the preprocessing scaler. In particular, we use


$$\begin{array}{l} S^{-1}=\text{Id},\;w_{t}=1+|y_{t}|, \end{array}$$

$$L=\frac{1}{T}\sum_{t=1}^{T}\left(1+|y_{t}|\right){\left(y_{t}-{\hat{y}}_{t}\right)}^{2}.$$

Weight depends on the magnitude of the true target. It improves training stability by ensuring that the model emphasizes the large peaks and strong seasonal fluctuations characteristic of influenza case counts.

To assess predictive performance independently of the training loss, we also evaluate model performance using standard error metrics.

- Mean absolute error (MAE).

$$\begin{array}{l} \text{MAE}=\frac{1}{T}\overset{T}{\underset{t=1}{\sum }}|y_{t}-{ŷ}_{t}|. \end{array}$$

- Root mean squared error (RMSE).

$$\begin{array}{l} \text{RMSE}=\sqrt{\frac{1}{T}\overset{T}{\underset{t=1}{\sum }}{\left(y_{t}-{ŷ}_{t}\right)}^{2}}. \end{array}$$

- Coefficient of determination (*R*^2^).

$$\begin{array}{l} {\text{R}}^{2}=1-\frac{\sum_{t}{\left(y_{t}-{ŷ}_{t}\right)}^{2}}{\sum_{t}{\left(y_{t}-ȳ\right)}^{2}},\;ȳ=\frac{1}{T}\overset{T}{\underset{t=1}{\sum }}y_{t}. \end{array}$$

The model is trained and validated using the weighted MSE loss, meaning that this loss function governs both how the model updates its parameters during training and how its performance is assessed on the validation set.

Because BPTT involves repeated jacobian products across many time steps, gradients may shrink or grow exponentially, leading to vanishing or exploding gradients. This limits the ability of basic RNNs to learn long-range dependencies, motivating gated architectures.

### 3.2 Long Short-Term Memory (LSTM) networks

LSTMs introduce gating mechanisms to regulate information flow and stabilize gradients. At time *t*, the forget, input, and output gates are Bengio et al. (2017):


$$\begin{array}{l} f_{t}=\sigma \left(W_{f}x_{t}+U_{f}h_{t-1}+b_{f}\right),\\ i_{t}=\sigma \left(W_{i}x_{t}+U_{i}h_{t-1}+b_{i}\right),\\ o_{t}=\sigma \left(W_{o}x_{t}+U_{o}h_{t-1}+b_{o}\right). \end{array}$$

The candidate cell state is


$$\begin{array}{l} {\tilde{c}}_{t}=\tanh\left(W_{c}x_{t}+U_{c}h_{t-1}+b_{c}\right), \end{array}$$

and the cell and hidden states update as:


$$\begin{array}{l} c_{t}=f_{t}⊙c_{t-1}+i_{t}⊙{\tilde{c}}_{t},\\ h_{t}=o_{t}⊙\tanh\left(c_{t}\right). \end{array}$$

The linear path through *c*_*t*_ mitigates vanishing gradients and enables long-term memory.

### 3.3 Gated Recurrent Unit (GRU)

GRUs simplify LSTMs while retaining gating behavior. The reset and update gates are Annadurai et al. (2025), Dey and Salem (2017):


$$\begin{array}{l} r_{t}=\sigma \left(W_{r}x_{t}+U_{r}h_{t-1}\right),\\ z_{t}=\sigma \left(W_{z}x_{t}+U_{z}h_{t-1}\right). \end{array}$$

The candidate hidden state is


$$\begin{array}{l} {\tilde{h}}_{t}=\tanh\left(W_{h}x_{t}+U_{h}\left(r_{t}⊙h_{t-1}\right)\right), \end{array}$$

and the hidden state update is a convex combination:


$$\begin{array}{l} h_{t}=\left(1-z_{t}\right)⊙h_{t-1}+z_{t}⊙{\tilde{h}}_{t}. \end{array}$$

The update gate controls how much past information is retained, while the reset gate determines how much of the previous state contributes to the candidate.

### 3.4 Transformer self-attention mechanism

Transformers eliminate recurrence and instead use self-attention to model dependencies across all time steps in parallel. Given input embeddings, the model constructs query, key, and value matrices (Vaswani et al., 2017):


$$\begin{array}{l} Q=XW_{Q},\;K=XW_{K},\;V=XW_{V}. \end{array}$$

#### 3.4.1 Scaled dot-product attention

Attention scores are computed as:


$$\begin{array}{l} A=\text{softmax}\left(\frac{QK^{⊤}}{\sqrt{d_{k}}}\right), \end{array}$$

where *d*_*k*_ is the key dimension. The attention output is:


$$\begin{array}{l} \text{Attention}\left(Q,K,V\right)=AV. \end{array}$$

#### 3.4.2 Multi-head attention and encoder block

Multiple attention heads learn different relational subspaces:


$$\begin{array}{l} \text{MHA}\left(X\right)=\left[H_{1}|H_{2}|\ldots |H_{h}\right]W_{O}. \end{array}$$

**Figure 1:**
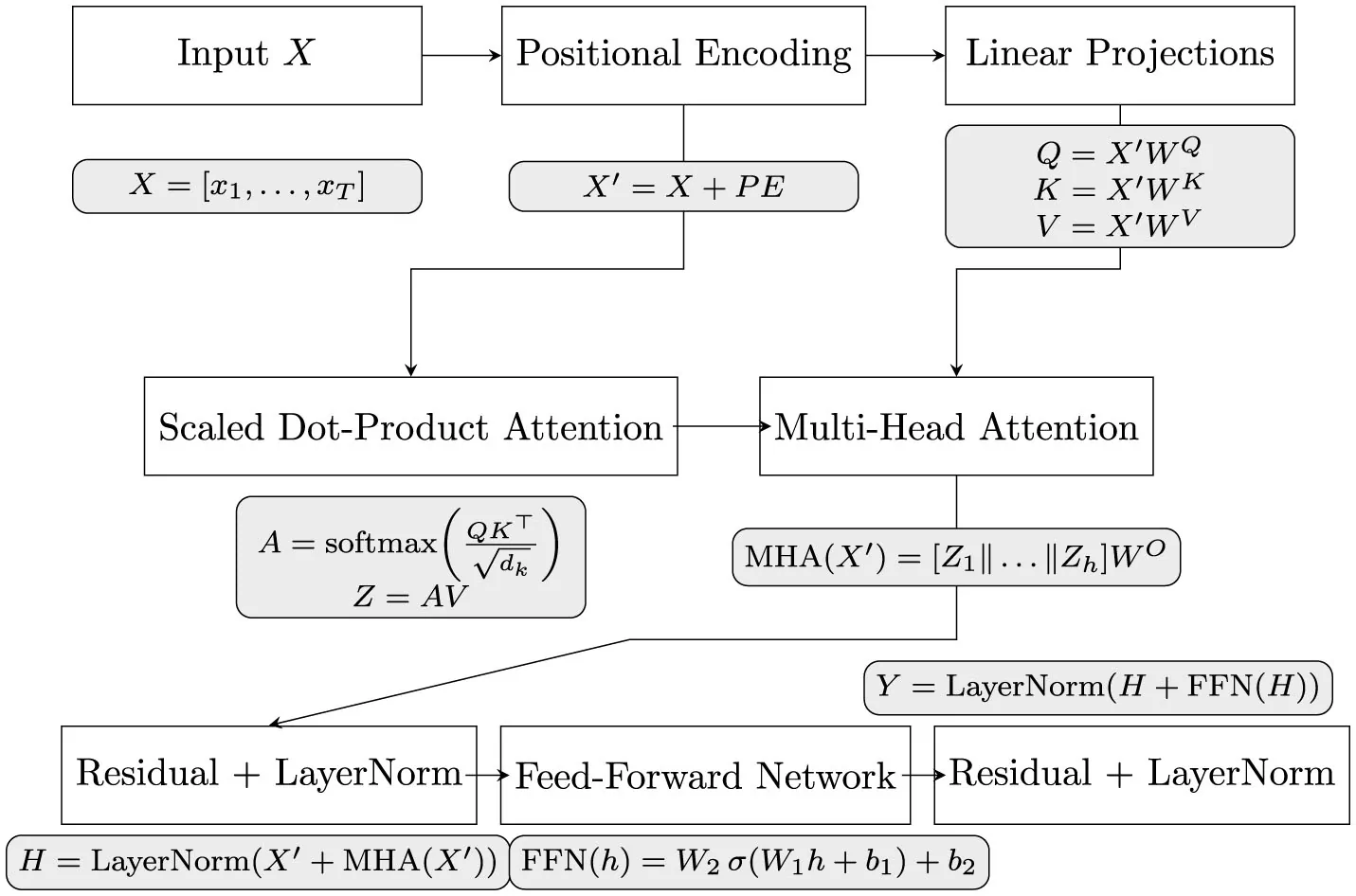
A transformer encoder block illustrating multi-head attention, residual connections, layer normalization, and feed-forward network.

Each encoder layer applies multi-head attention, residual connections, layer normalization, and a position-wise feed-forward network, enabling the model to capture long-range dependencies efficiently and in parallel. Figure 1 shows a transformer encoder block illustrating multi-head attention, residual connections, layer normalization, and feed-forward network.

## 4 Methodology

In this study, we evaluate the performance of three deep learning architectures (LSTM, GRU, and transformer encoder models) for forecasting monthly influenza incidence in Ontario. The methodological framework consists of data acquisition, preprocessing, model construction, training, and evaluation, to ensure a controlled comparison across architectures. We ensure a controlled architecture-level comparison by using the same batch size, number of epochs, training windows, optimizer, loss function, number of layers, and dropout rate across all three deep learning models.

The dataset is comprised of laboratory-confirmed influenza cases in Ontario from January 2014 to November 2025 obtained from public health Ontario (Public Health Ontario, 2026) and mean daily temperatures recorded at the Toronto city weather station, obtained from environment and climate change Canada (Environment and Climate Change Canada, 2026). The mean daily temperatures are aggregated into monthly averages. After temporal alignment, the combined dataset spans January 2014 to November 2025, yielding 143 monthly observations.

**Figure 2:**
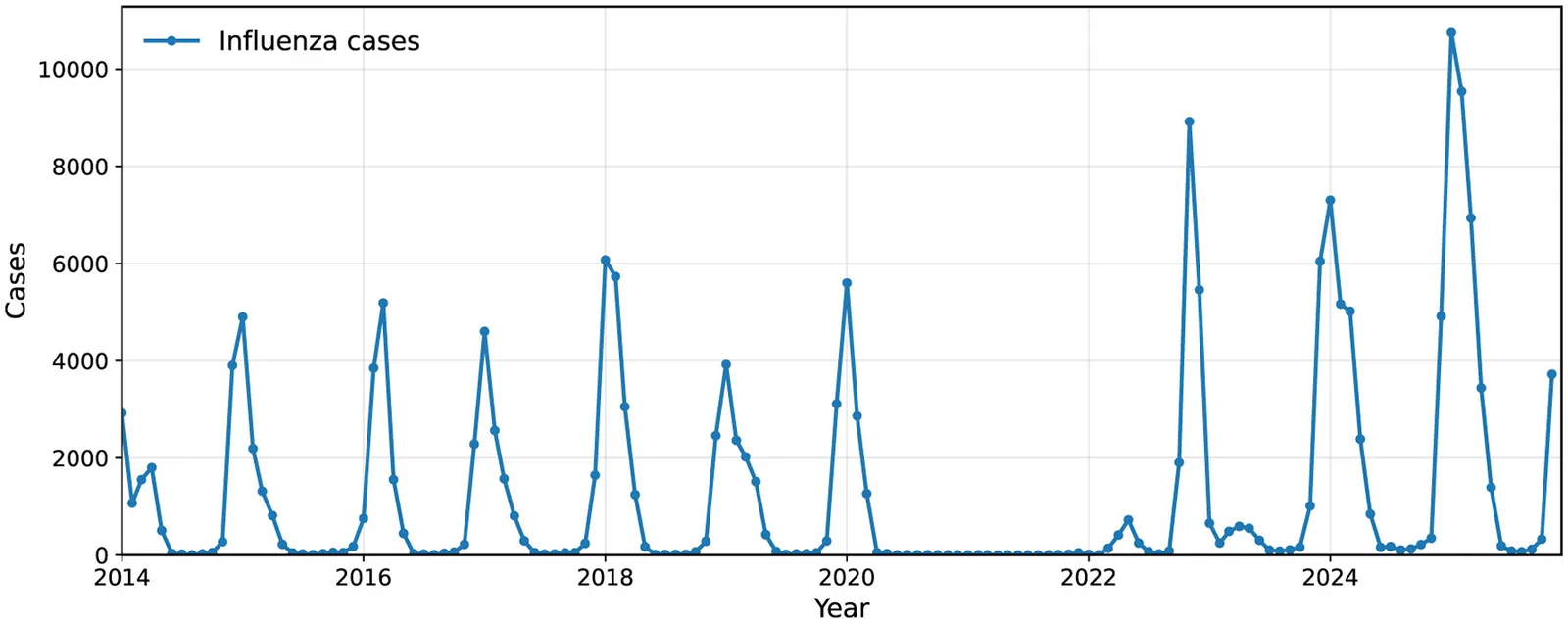
Monthly laboratory-confirmed influenza cases in Ontario from January 2014 to November 2025.

Before model training, we standardize the data using *z*-score normalization (standard scaler, Equation 1) computed on the training set. This ensures that both variables, i.e., temperature and influenza counts, are placed on a comparable scale for multivariate learning. We construct supervised learning windows by mapping the previous 12 months of data to a one-step-ahead prediction target. We divide the data chronologically into training (January 2014–July 2017), validation (August 2017–July 2020), and test (August 2022–November 2025) sets. The period from August 2020 to July 2022 is excluded because influenza circulation was extremely low during this interval due to COVID-19 restrictions, resulting in near-zero influenza activity and insufficient signal for model training or evaluation. Figure 2 shows the full influenza time series used for model evaluation.

We use the continuous timeline (143 months) to construct the input windows, while the 24-month COVID period (August 2020–July 2022) is excluded from all train/validation/test splits. The modeling dataset hence contains 43 training points, 36 validation points, and 40 test points (119 total). With a sequence length of 12, each model input consists of a 12-month window used to predict the following month. This produces 31 training windows, and with a batch size of 12, each epoch performs three gradient updates.

All three deep learning models are trained under a unified configuration to ensure comparability across architectures. The LSTM and GRU models each consist of a single recurrent layer with 64 units, followed by a dense output layer, while the transformer model uses a single encoder block with multi-head attention and a feed-forward network. The batch size, number of epochs, optimizer settings, and weighted MSE loss were kept identical across models to maintain consistent training. The Table 1 below lists the hyperparameters involved in the training and validation process of the three deep learning models. The hyperparameters are selected through manual tuning across the search space given by the number of layers {2, 4, 6, 8, 10, 12}, batch sizes {12, 16, 32, 64, 128}, learning rates {0.1, 0.01, 0.001, 10^−4^, 10^−5^, 10^−6^}, dropout rates {0.0, 0.1, 0.2, 0.3, 0.4, 0.5}, and activation functions {ReLU, sigmoid, tanh} to identify the configuration that minimized validation MSE.

**Table 1:** Hyperparameter specification for the LSTM, GRU, and transformer models.

Hyperparameter	LSTM	GRU	Transformer
Units	64	64	*d*_model_ = 64
Layers	1	1	1 encoder block
Batch size	12	12	12
Activation	tanh	tanh	tanh (FFN)
Epochs	50	50	50
Learning rate	0.001	0.001	0.001
Dropout	0.1	0.1	0.1
Recurrent dropout	None	None	None
Optimizer	Adam	Adam	Adam
Loss function	Weighted MSE	Weighted MSE	Weighted MSE

Because the dataset contains only 43 training points (31 training windows), increasing the transformer depth beyond a single encoder layer results in unstable training and degraded predictive performance. Deeper architectures require substantially more data to learn meaningful temporal representations, and with small dataset, they tend to overfit or fail to converge. For this reason, the transformer is kept at one encoder layer, which provides the most reliable and interpretable results for this forecasting task.

We evaluate the model performance using unscaled error metrics. The mean absolute error (MAE), root mean squared error (RMSE), and the coefficient of determination (*R*^2^) are computed for the both validation and test set. Quantitative evaluation is complemented by visual inspection of predicted vs. observed trajectories to assess peak timing, amplitude, and overall seasonal alignment.

This methodological design enables a systematic comparison of recurrent and attention-based architectures under both stable and disrupted epidemiological regimes, providing insight into their robustness, generalization, and suitability for real-world influenza forecasting.

## 5 Results and discussion

The three neural architectures (LSTM, GRU, and transformer) are evaluated on monthly influenza cases in Ontario using error metrics across the test periods. Models are trained on January 2014–July 2017 and evaluated on a pre-COVID validation regime (August 2017–July 2020) and a post-COVID test regime (August 2022–November 2025), during which influenza dynamics exhibited substantial non-stationarity. The multivariate input included influenza cases and monthly mean temperature, reflecting both intrinsic epidemic dynamics and environmental drivers.

### 5.1 Model performance

Table 2 presents the final performance of all models using influenza cases and temperature as inputs. Across the deep learning architectures, none of the models achieve strong predictive accuracy in the post-COVID period. All three (LSTM, GRU, and Transformer) exhibit high RMSE values (2,973.74 ± 51.33, 2,822.38 ± 94.11, and 2,941.00 ± 77.47, respectively) and low *R*^2^ scores (0.038 ± 0.033, 0.133 ± 0.058, and 0.059 ± 0.050), indicating that only a small fraction of the variance in post-COVID influenza incidence is explained. A consistent pattern across all deep learning models is the systematic under-prediction of post-COVID influenza peaks. While the recurrent models capture the timing and general seasonal structure, they fail to reproduce the magnitude of the observed peaks. This behavior is characteristic of a structural break, i.e., the post-COVID influenza regime exhibits elevated and irregular incidence patterns none of which are represented in the pre-COVID training data. As a result, the models extrapolate from outdated seasonal dynamics and underestimate the true post-COVID burden. The classical baselines contextualize this difficulty. The persistence model achieves the lowest RMSE (2,359.18), outperforming all deep learning models despite its simplicity. The seasonal naïve and SARIMAX models also perform competitively (RMSE = 2,829.78 and 2,702.88), and the Random Forest achieves RMSE = 2,683.17 ± 13.47, surpassing all neural architectures. These results highlight that, under severe non-stationarity and limited data, models with strong inductive biases or lower variance can outperform flexible deep learning models that rely heavily on stable historical patterns.

**Table 2:** Final model performance using cases and temperature input features.

Model	MAE	RMSE	*R* ^2^	95% CI (RMSE)
LSTM	1,757.17 ± 29.34	2,973.74 ± 51.33	0.038 ± 0.033	(2,940.21, 3,007.28)
GRU	1,662.66 ± 57.53	2,822.38 ± 94.11	0.133 ± 0.058	(2,760.90, 2,883.87)
Transformer	2,164.42 ± 183.22	2,941.00 ± 77.47	0.059 ± 0.050	(2,890.03, 2,990.92)
Seasonal Naïve	1,589.58 ± 0.00	2,829.78 ± 0.00	0.129 ± 0.00	(2,829.78, 2,829.78)
Persistence	1,447.65 ± 0.00	2,359.18 ± 0.00	0.395 ± 0.00	(2,359.18, 2,359.18)
SARIMAX	1,716.39 ± 0.00	2,702.88 ± 0.00	0.206 ± 0.00	(2,702.88, 2,702.88)
Random forest	1,624.47 ± 7.75	2,683.17 ± 13.47	0.22 ± 0.01	(2,674.82, 2,691.51)
**Deep learning model significance tests (paired** ***t*****-test on RMSE)**				
**Model comparison**			***p*** **-value**	
LSTM vs. GRU			2.75 × 10^−5^	
LSTM vs. transformer			0.225	
GRU vs. transformer			0.010	

Metrics are reported as mean ± standard deviation over 10 seeds = [0, 1, 2, 3, 4, 10, 42, 100, 123, 999], with 95% confidence intervals (CI).

The paired *t*-tests conducted on RMSE values show that the LSTM and transformer do not differ significantly (*p* = 0.225), suggesting that their RMSE performance is statistically indistinguishable. The GRU and LSTM differ significantly, with a *p*-value of 2.75 × 10^−5^, indicating that their prediction errors are not statistically comparable. A significant difference is also observed between the GRU and Transformer models (*p* = 0.010), further showing that these architectures produce meaningfully different error distributions. The GRU's comparatively more stable behavior across the regime shift arises from its simplified gating structure, which reduces parameter count and mitigates the compounding of errors that LSTMs and Transformers experience when learning from limited, noisy, and non-stationary epidemiological sequences.

### 5.2 Influenza and temperature trends

**Figure 3:**
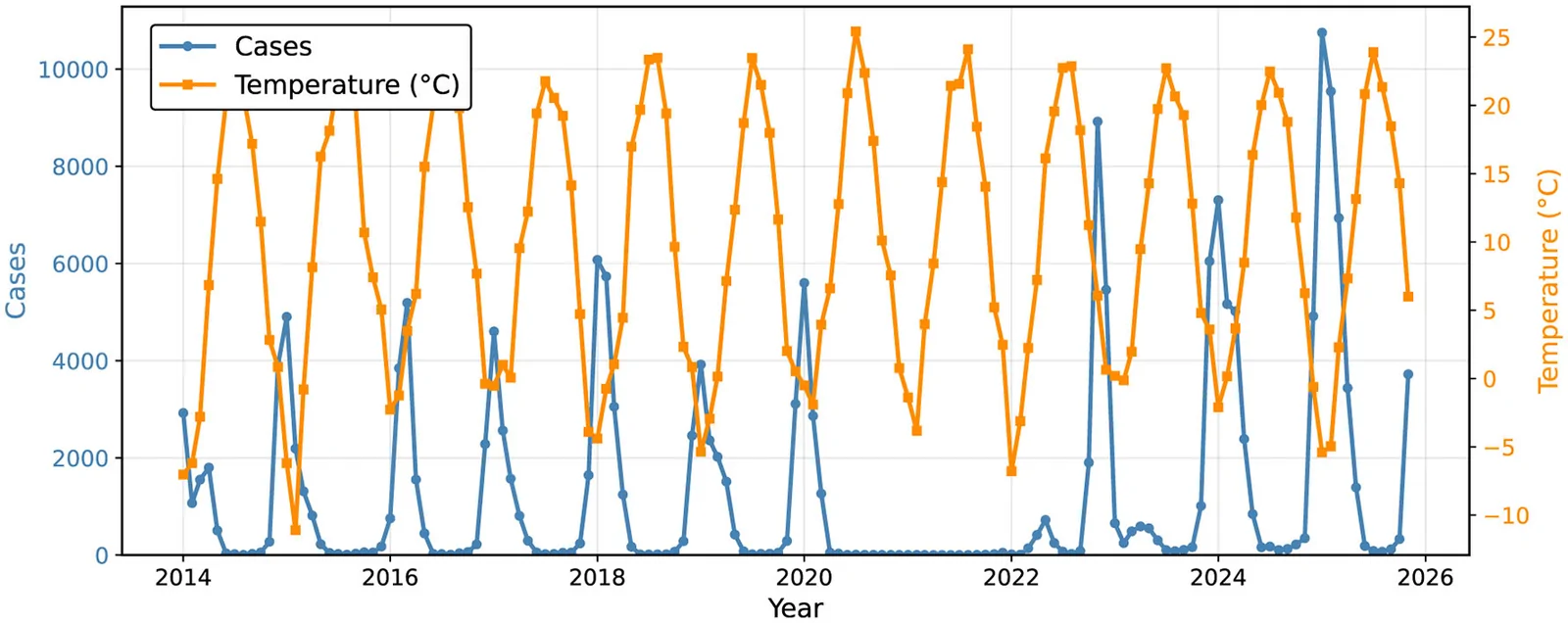
Joint visualization of influenza incidence and temperature, capturing the seasonal cycles and environmental variability.

Figure 3 shows joint visualization which highlights the seasonal variation between influenza incidence and temperature. The influenza time series exhibits a clear annual seasonal pattern, with sharp winter peaks followed by low summer incidence. Peak magnitudes vary substantially across years, with notably elevated and irregular post-COVID seasons reflecting disrupted transmission dynamics. The temperature series shows the expected inverse seasonal relationship, with colder months aligning with higher influenza activity, highlighting the environmental coupling captured in our multivariate models.

As shown in Table 3 below, the temperature ablation demonstrates that the effect of incorporating temperature varies across models, with some architectures exhibiting modest changes and others benefiting more substantially. The LSTM shows small but consistent improvements when temperature is added, whereas the GRU displays limited gain, showing slightly reduced performance with temperature added. In the cases-only setting, the transformer exhibits unstable training behavior and fails to converge, reflected in a negative *R*^2^ value of −0.088 and a standard deviation exceeding the mean ±0.228. However, incorporating temperature improves stability and accuracy, reducing MAE from 2, 692.98 ± 381.21 to 2, 164.42 ± 183.22 and RMSE from 3, 148.23 ± 321.85 to 2, 941.00 ± 77.47, while increasing *R*^2^ to 0.059 ± 0.050. This pattern is consistent with the model's reliance on attention mechanisms, which lack a built-in temporal structure and therefore benefit from the additional exogenous signal. Among the classical baselines, SARIMAX improves over SARIMA, and the Random Forest also shows clear gains when temperature is included. Overall, while the magnitude of improvement differs across models, the results indicate that incorporating temperature can provide meaningful and sometimes substantial benefits, particularly for architectures such as the Transformer that are well-positioned to use exogenous variables.

**Table 3:** Temperature ablation across all models.

Model	Metric	Inputs	
		Influenza cases	Influenza cases, temperature
LSTM	MAE	1,769.83 ± 23.35	1,757.17 ± 29.34
	RMSE	2,987.06 ± 61.91	2,973.74 ± 51.33
	*R* ^2^	0.030 ± 0.040	0.038 ± 0.033
GRU	MAE	1,629.09 ± 51.00	1,662.66 ± 57.53
	RMSE	2,744.23 ± 97.96	2,822.38 ± 94.11
	*R* ^2^	0.180 ± 0.059	0.133 ± 0.058
Transformer	MAE	2,692.98 ± 381.21	2,164.42 ± 183.22
	RMSE	3,148.23 ± 321.85	2,941.00 ± 77.47
	*R* ^2^	–0.088 ± 0.228	0.059 ± 0.050
SARIMA/ SARIMAX	MAE	1,632.19 ± 0.00	1,716.39 ± 0.00
	RMSE	2,820.50 ± 0.00	2,702.88 ± 0.00
	*R* ^2^	0.135 ± 0.00	0.206 ± 0.00
Random forest	MAE	1,700.73 ± 5.71	1,624.47 ± 7.75
	RMSE	2,797.19 ± 7.47	2,683.17 ± 13.47
	*R* ^2^	0.15 ± 0.00	0.22 ± 0.01

Metrics are reported as mean ± standard deviation over 10 seeds = [0, 1, 2, 3, 4, 10, 42, 100, 123, 999].

### 5.3 Model behavior over time

**Figure 4:**
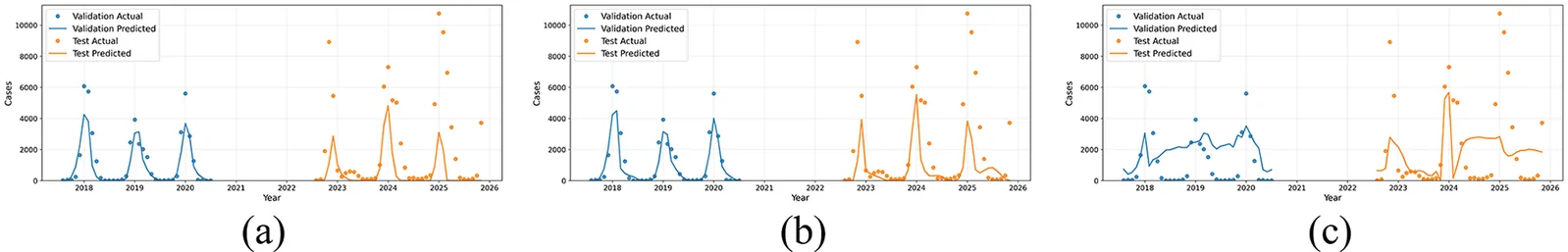
Model performance visualizations. **(a)** LSTM predictions vs. actuals. **(b)** GRU predictions vs. actuals. **(c)** Transformer predictions vs. actuals.

**Figure 5:**
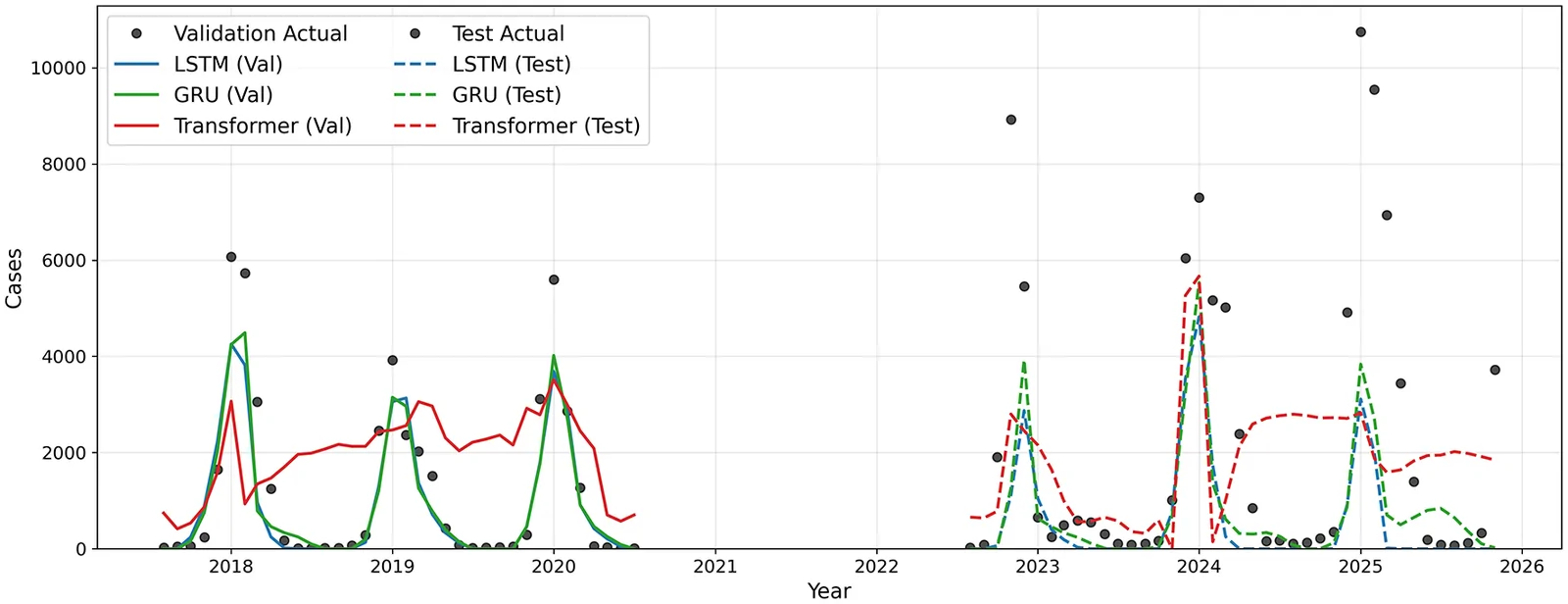
A comparison plot for LSTM, GRU and transformer predictions across the validation and test periods. The plot highlights the ability of RNN and transformer model to capture seasonal peaks.

**Figure 6:**
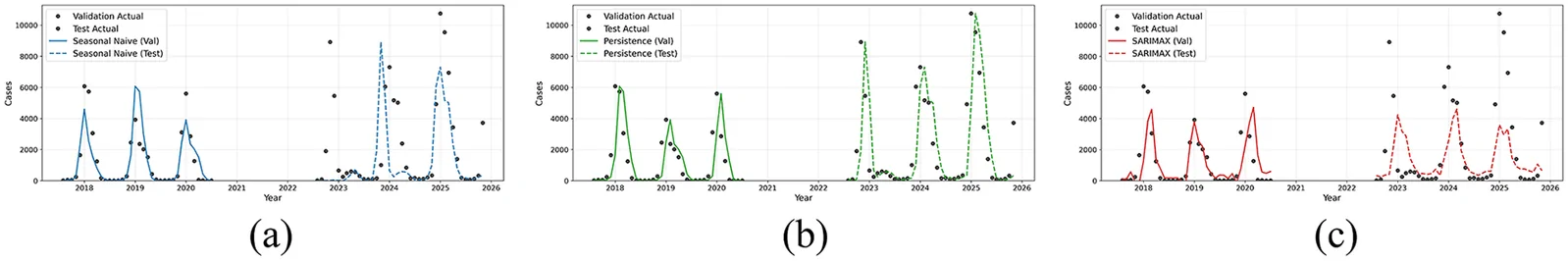
Model performance visualizations. **(a)** Seasonal naïve predictions vs. actuals. **(b)** Persistence predictions vs. actuals. **(c)** SARIMAX predictions vs. actuals.

**Figure 7:**
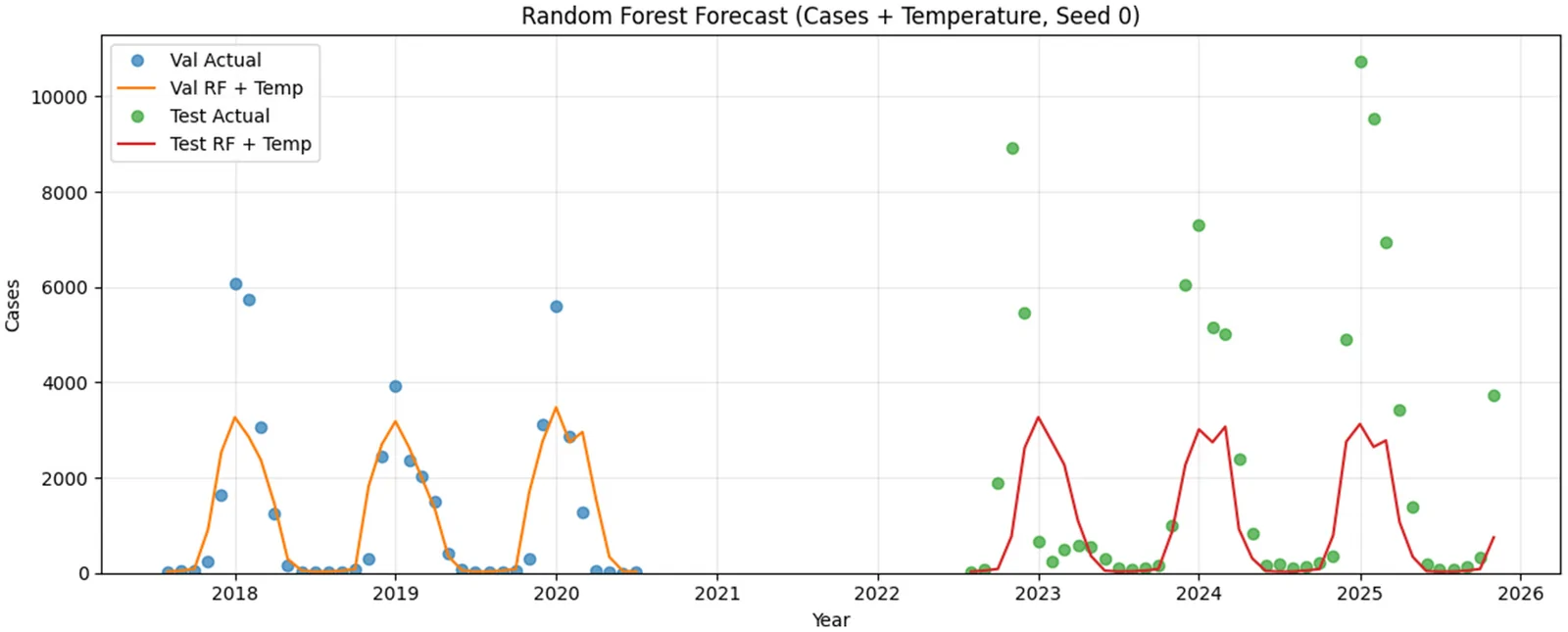
Random forest predictions vs. actuals.

The figures below illustrate how each forecasting model behaves across the pre-COVID validation period and the post-COVID test period, highlighting how the regime shift affects the model performance. Figure 4 shows that, during the test period, the deep learning models underpredict the peaks. Both LSTM and GRU closely tracked seasonal peaks, with the GRU producing smoother trajectories. The transformer underfits peak magnitude, but captures some peaks. Figure 5 compares predicted and observed influenza cases. Although the deep learning models capture the right timing of influenza incidence and are accurate in showing the influenza seasonality, the regime shift post-COVID causes actual cases to rise beyond the model's predictions. Figures 6, 7 present the corresponding forecasts for the classical baselines. The persistence model closely follows observed peaks and achieves the strongest test performance, reflecting its robustness under structural breaks. The SARIMA/SARIMAX and the random forest models reproduce the broad seasonal pattern but underestimate peak height, while the seasonal naïve also captures peak timing and amplitude more effectively than the neural network models.

## 6 Conclusion

This work examines the performance of classical and deep learning models for forecasting influenza incidence during post-COVID period under limited real-world data. Deep learning architectures such as the LSTM, GRU, and transformer capture the timing and general seasonal structure of influenza activity but consistently under-predicted the magnitude of post-COVID peaks. Classical baselines, including the persistence model, seasonal naïve model, SARIMAX, and Random Forest, performed competitively and in several cases outperform the neural network models. These results highlight the profound impact of the COVID-19 pandemic on influenza transmission dynamics and underscore the challenge of forecasting in the presence of structural breaks. These results do not indicate a failure of deep learning models, but rather illuminate the profound instability of the post-COVID influenza regime. The consistent under-prediction of peak magnitudes across all neural architectures reflects the fact that the underlying epidemiological process has shifted in ways that are not represented in the pre-COVID training data. In this context, the competitive performance of classical baselines underscores an important methodological insight that with limited, non-stationary data marked by a regime shift, models with strong inductive biases or lower variance can outperform more flexible architectures that rely on stable historical patterns. These findings highlight the need for models that explicitly account for regime shifts, exogenous variables, and non-stationarity, pointing toward promising directions for future work in post-pandemic forecasting. The improvement observed when temperature is included as an exogenous variable, particularly for the transformer, demonstrates that integrating environmental and behavioral covariates meaningfully enhances model stability under non-stationarity. As surveillance systems continue to evolve in the post-pandemic era, models that can dynamically incorporate new information and adjust to shifting epidemiological conditions will be essential. Therefore, with disease dynamics shifting continuously, future influenza forecasting systems would benefit from incorporating adaptive mechanisms such as online learning, regime-switching models, or hybrid mechanistic-machine learning approaches to better accommodate non-stationarity and the limited data constraints highlighted by our experiments.

## Data Availability

Publicly available datasets were analyzed in this study. This data can be found here: the data that support the findings of this study are openly available from Public Health Ontario at https://www.publichealthontario.ca/en/Diseases-and-Conditions/Infectious-Diseases/Respiratory-Diseases, and from Environment and Climate Change Canada at https://climate.weather.gc.ca/historical_data/search_historic_data_e.html.
